# M^3^Site: multiclass multimodal learning for protein active site identification and classification

**DOI:** 10.1093/bib/bbaf590

**Published:** 2025-11-12

**Authors:** Song Ouyang, Yong Luo, Huiyu Cai, Kehua Su, Fei Liao, Na Zhan, Huangxuan Zhao, Tailang Yin, Lin Zhao, Dongjing Shan

**Affiliations:** Renmin Hospital of Wuhan University, Zhang Road and Jiefang Road, Wuhan, Hubei 430060, China; School of Computer Science, National Engineering Research Center for Multimedia Software, Wuhan University, Bayi Road, Wuhan, Hubei 430072, China; School of Computer Science, National Engineering Research Center for Multimedia Software, Wuhan University, Bayi Road, Wuhan, Hubei 430072, China; BioGeometry, North Haidian 2nd Street, Beijing 100083, China; Mila - Québec AI Institute, Rue Saint-Urbain, Montréal, Québec H2S 3H1, Canada; Department of Computer Science and Operations Research, Université de Montréal, Bd Édouard-Montpetit, Montréal, Québec H3T 1J4, Canada; School of Computer Science, National Engineering Research Center for Multimedia Software, Wuhan University, Bayi Road, Wuhan, Hubei 430072, China; Renmin Hospital of Wuhan University, Zhang Road and Jiefang Road, Wuhan, Hubei 430060, China; Renmin Hospital of Wuhan University, Zhang Road and Jiefang Road, Wuhan, Hubei 430060, China; School of Computer Science, National Engineering Research Center for Multimedia Software, Wuhan University, Bayi Road, Wuhan, Hubei 430072, China; Renmin Hospital of Wuhan University, Zhang Road and Jiefang Road, Wuhan, Hubei 430060, China; Peking Union Medical College Hospital, No. 1 Shuaifuyuan Wangfujing Dongcheng District, Beijing 100730, China; Southwest Medical University, Zhongshan Road, Luzhou, Sichuan 646000, China

**Keywords:** protein, active site identification, multimodal learning, multiclass classification

## Abstract

Accurately identifying and classifying protein active sites is crucial for understanding protein mechanisms, drug design, and synthetic biology. Current methods often rely on binary classification and single-modal data, limiting their scope. To address these limitations, we propose M$^{3}$Site, a multimodal framework that integrates protein sequence embeddings, structural graph representations, and functional text annotations for residue-level, multiclass active site prediction. Built upon a curated dataset of 25 883 proteins sourced from UniProt and AlphaFold2, M$^{3}$Site leverages pretrained protein language models, equivariant graph neural networks, and biomedical language models for feature extraction. The function informed cross-attention module enables cross-modal feature fusion, while the adaptive weighted fusion mechanism balances modality contributions. A compound loss function tackles class imbalance, ensuring robust performance. Experimental results show M$^{3}$Site significantly outperforms existing models, and an interactive application has been developed to enhance its practical utility for predictions and visualizations. The dataset, source code for experiments, and interactive application are publicly available at https://github.com/Gift-OYS/M3Site.

## Introduction

Proteins are central to virtually all biological processes, with their function often dictated by specific active sites, i.e. discrete regions where biochemical reactions or molecular interactions occur. Accurate identification of these active sites, along with their functional roles, is critical to advance our understanding of protein function, guide rational drug design, and engineer novel biomolecules [1]. Traditional methods for active site identification rely heavily on experimental techniques, such as X-ray crystallography [2], nuclear magnetic resonance spectroscopy [3], and cryo-electron microscopy [4]. These approaches are often labor-intensive, time-consuming, and expensive, limiting their scalability for large-scale studies. Early computational methods primarily relied on rule-based pattern-matching approaches, such as Discern [5], or conventional machine learning models like Random Forest and Support Vector Machine, trained on hand-crafted features [6]. However, these methods often suffered from limited generalizability, implementation complexity, and suboptimal predictive performance. In addition, they failed to capture the complex relationship between sequence, structure, and functional information.

With the advent of deep learning, there has been a paradigm shift toward data-driven methods that automatically learn hierarchical representations from protein sequence and structural data. Notable progress includes two key approaches: structure-based models utilizing graph neural networks like IEConv [7], GearNet [8], and TransGCN [9], and sequence-based models powered by protein language models (PLM) such as ProtTrans [10], ProteinBERT [11], TargetCLP [12], and D2Deep [13]. These methods have demonstrated remarkable success in predicting protein-level properties, such as biological functions, stability, or binding affinity. However, these approaches primarily operate at the protein level and often overlook residue-level classification tasks, which remains challenging because of the lack of high-quality annotated datasets. Although some efforts have been made to identify binding sites with ligands, nucleic acid [14], and gene sequences such as DeepSurf [15], DeepProSite [16], EGPDI [17], and ULDNA [18], these methods typically focus on binary classification tasks. They often do not provide information on the functional specificity of the identified sites, which limits their utility in understanding more complex biological meanings.

Recent studies partly fill these gaps but still remain limited. For instance, CVPM [19] was proposed for residue-level identification of human lysine-acetylation sites by coupling handcrafted sequence–structure features. But it tackles a binary modification problem on a relatively small, single-species dataset, and combines sequence and structure into a unified feature vector, lacking an explicit mechanism to capture cross-modal interactions. On the other hand, oral_voting_transfer [20] ensemble was introduced to migrate knowledge from Golgi proteins to five oral bacterial species for function and active site prediction. But it also focus on single species using single-sequence embeddings, thereby failing to localize individual residues or differentiate multiple functional roles within a protein.

**Figure 1:**
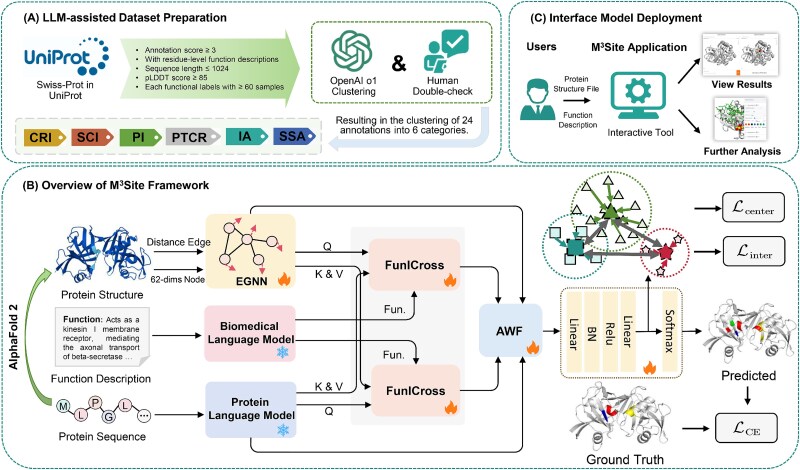
Overview of our work: (A) LLM-assisted dataset preparation from Swiss-Prot in UniProt via filtering, semantic annotation clustering into six categories, and manual verification; (B) M^3^Site framework that jointly encodes structural, sequential, and textual features using EGNN, PLM, and BLM, integrates function information via FunICross module, fuses features with AWF mechanism, and uses a composite loss for training; (C) interactive deployment allowing users to input protein structures and function descriptions for residue-level prediction visualization and further analysis.

In our previous work, MMSite [21], we addressed the binary classification problem of identifying active sites in proteins by leveraging sequence data and multi-attribute text descriptions. However, it was inherently limited by its difficulty to capture comprehensive textual information and the lack of structural data, which restricted its ability to fully understand the spatial context of the active sites. Moreover, MMSite did not explore the functional heterogeneity of these sites. To overcome these limitations, we developed M$^{3}$Site, an improved framework that goes beyond mere active site identification, enabling the classification of the functional roles of these sites (shown as Fig. 1).

M$^{3}$Site leverages a multimodal approach, integrating three distinct types of information—protein sequence, structure, and functional text descriptions. To support this effort, we constructed a high-quality dataset of 25 883 proteins, including residue-level functional annotations derived from UniProt [22, 23] and structural data sourced from the AlphaFold database [24, 25]. Functional labels of active sites were refined into six categories by large language models (LLMs) and expert validation, ensuring both accuracy and interpretability. In M$^{3}$Site, for structural data, we designed a 62-dimensional vector to encapsulate the physicochemical properties of each residue, constructed spatial graphs, and extracted node-level features using equivariant graph neural networks (EGNN) [26]. Sequence features were extracted using pretrained PLM, while functional text descriptions were encoded with biomedical language models (BLM). To effectively aggregate these heterogeneous representations, we designed the FunICross module, which employs symmetric cross-attention mechanisms to integrate information across modalities. Additionally, we introduced the adaptive weighted fusion (AWF) mechanism to dynamically combine the fused multimodal features with their original representations. To address the challenge of extreme class imbalance inherent to active site identification, we designed a compound loss function that incorporates both intra-class compactness and inter-class separability, enhancing the discriminative power of the model. Comprehensive experiments demonstrate that M$^{3}$Site significantly outperforms existing protein representation learning baselines. Finally, to promote the practical applicability of our work, we developed an interactive interface using Gradio [27], allowing researchers to predict, visualize, and analyze active site functionalities in a user-friendly manner. We believe that M$^{3}$Site and its accompanying dataset and tool make an important step forward in protein active site analysis.

## Materials and methods

### Benchmark dataset

Given the scarcity of high-quality datasets with residue-level, multiclass functional annotations, we constructed a large-scale, high-quality dataset by systematically curating data from multiple sources. We began with collecting protein sequences from the Swiss-Prot in UniProt, deposited before 28 July 2024. To ensure the reliability of functional annotations for active sites, we filtered based on the following criteria: annotation score $\geq $3, precise textual descriptions specifying active site functions are available, and sequence lengths under 1024 amino acids. For structural data, we retrieved from the AlphaFold Protein Structure Database and filtered for structures with a pLDDT score $\geq $85 to ensure high structural confidence. To maintain generality and focus on functionally significant proteins, only functional labels associated with at least 60 samples were included.

To merge highly similar raw functional annotations, improve biological interpretability, and mitigate class imbalance, we employed OpenAI’s o1 model [28] to cluster raw functional annotations. Initially, the model was instructed via API to group annotations into 5–10 biologically meaningful categories, provide detailed descriptions, and flag ambiguous cases (see Supplementary Note S1 for details). After that, domain experts manually refined the results through rigorous review, yielding six consolidated functional categories: Covalent Reaction Intermediates (CRI), Sulfur-containing Covalent Intermediates (SCI), Phosphorylated Intermediates (PI), Proton Transfer & Charge Relay Systems (PTCR), Isomerization Activity (IA), and Substrate-specific Activities (SSA) (see Supplementary Table S1 for more details). These categories serve as the classification targets for our framework.

To prevent potential information leakage caused by sequence similarity, we employed MMSeqs2 [29] to cluster sequences based on pairwise similarity. Specifically, we clustered the sequences at similarity thresholds of 10%, 30%, 50%, 70%, and 90%, following MMSite [21]. In each cluster, 20 samples were randomly selected to ensure the diversity. The final dataset was divided into training, validation, and testing sets with a ratio of 8:1:1 (see Supplementary Table S2). Table 1 shows the number of amino acids corresponding to each category label under different thresholds.

**Table 1:** Statistics of amino acid counts for each category label under different clustering thresholds

Category	10%	30%	50%	70%	90%
CRI	588	697	1729	2885	3088
SCI	1122	1202	2509	3713	4276
PI	472	555	1155	2273	2622
PTCR	6942	7830	14 163	21 235	23 418
IA	20	20	130	152	153
SSA	80	198	586	716	716
None	$\sim $ 3M	$\sim $ 3.3M	$\sim $ 6.1M	$\sim $ 9M	$\sim $ 10M

Note: “None” denotes amino acids that are nonactive sites.

### Task formulation

Denote a protein $\mathcal{P}=\{\mathbf{S},\mathcal{G}(\mathcal{V},\mathcal{E}),\mathbf{T}\}$, where $\mathbf{S}\in \mathbb{R}^{L\times 20}$ represents the amino acid sequence encoded as one-hot vectors for $L$ residues, $\mathcal{G}=\left (\mathcal{V},\mathcal{E}\right )$ is a graph representation of the protein’s 3D structure, and $\mathbf{T}$ is a text description of the protein’s function. The task of active site prediction assigns a residue-level label $y_{i}\in \left \{0,1,\ldots ,K-1\right \}$ to each amino acid $a_{i}\in \mathbf{S}$, where $y_{i}=0$ indicates a nonactive site and $y_{i}\in \left \{1,\ldots ,K-1\right \}$ specifies one of $K-1$ active site categories. Our task is to map the multimodal inputs to a probability distribution over classes for each residue ${\hat{\mathbf{y}}}_{i}=\left [{\hat{y}}_{i,0},{\hat{y}}_{i,1},\ldots ,{\hat{y}}_{i,K-1}\right ]$, satisfying $\sum _{k=0}^{K-1}{\hat{y}}_{i,k}=1$. The final predicted class is ${\hat{y}}_{i}=arg\max \limits _{k} {{\hat{y}}_{i,k}}$.

### Protein representation construction

To accurately predict protein active sites, we construct three complementary representations of the proteins: sequence, structure, and function text.

#### Sequence representation

The amino acid sequence $\mathbf{S}=\left \{s_{1},s_{2},\ldots ,s_{L}\right \}$ of a protein is first processed using a pretrained PLM to capture its contextual semantic information. Consequently, the pretrained PLM extracts high-dimensional embeddings of $\mathbf{S}$, resulting in: 


(1) \begin{align*}& \mathbf{F}_{\mathrm{seq}}=\mathrm{PLM}(\mathbf{S}) \in \mathbb{R}^{L\times d_{s}},\end{align*}

where $d_{s}$ is the sequence dimension.

#### Structure representation

To capture the structural and spatial properties of proteins, we represent the protein as a graph $\mathcal{G}=\left (\mathcal{V},\mathcal{E}\right )$, where each node $v_{i}\in \mathcal{V}$ corresponds to a residue $a_{i}$ and edges $e_{ij}\in \mathcal{E}$ connect residues based on spatial proximity. Then we employ the EGNN to extract node-level representations that encode both local residue features and global structural context. Specifically, each node is initialized with a feature vector $\mathbf{h}_{v_{i}}^{0}\in \mathbb{R}^{62}$ (see Supplementary Table S3), which is different from the 62-dims sequence-level feature of MEG-PPIS [30]. It includes 9-dims representing the protein’s secondary structure using one-hot encoding, 16-dims for the backbone and side-chain torsion angles ($\phi $, $\psi $, $\omega $, $\chi _{1}$, $\chi _{2}$,..., $\chi _{5}$) and their sine and cosine values, 7-dims for atomic features such as atomic mass, B-factor, side-chain status, electronic charge, hydrogen bonding, ring membership, and van der Waals radius, as well as 2-dims for hydrogen bond information derived from MDtraj [31]. We further include a 1-dim pseudo-position embedding to encode the position of each residue in the sequence, and 6-dims capture other amino acid properties, such as hydrophobicity, polarity, charge, pKa, volume, and mass. Furthermore, the one-hot encoding of the 21 amino acid types (including one unknown type) is included in the node feature vector. The edges in the graph are constructed based on spatial proximity between residues. An edge $e_{ij}$ exists between residues $v_{i}$ and $v_{j}$ if the Euclidean distance between their C$\alpha $ atoms is <8 Å, and the feature $a_{ij}$ is the distance. The 3D coordinates of the nodes are denoted as $\mathbf{X}^{0}=\left [\mathbf{x}_{1}^{0},\mathbf{x}_{2}^{0},\ldots ,\mathbf{x}_{L}^{0}\right ]\in \mathbb{R}^{L\times 3}$.

EGNN iteratively updates the node features $\mathbf{h}_{i}^{l}$ and coordinates $\mathbf{x}_{i}^{l}$ at each layer $l$ while preserving the geometric relationships inherent to the protein structure. The edge-wise message $\mathbf{m}_{ij}$ is computed using the function $\phi _{e}$ as follows: 


(2) \begin{align*}& \mathbf{m}_{ij}=\phi_{e}\left(\mathbf{h}_{i}^{l}, \mathbf{h}_{j}^{l}, \Vert x_{i}^{l}-x_{j}^{l} \Vert^{2},a_{ij}\right).\end{align*}

The node coordinates are updated using a new function $\phi _{x}$: 


(3) \begin{align*}& \mathbf{x}_{i}^{l+1}=\mathbf{x}_{i}^{l}+\left(M-1\right)^{-1}\sum_{j\neq i}{\left(\mathbf{x}_{i}^{l}-\mathbf{x}_{j}^{l}\right)\phi_{x}\left(\mathbf{m}_{ij}\right)},\end{align*}

where $M$ is the number of nodes in the graph. Simultaneously, the messages from all neighboring nodes are aggregated for each node: 


(4) \begin{align*}& \mathbf{m}_{i}=\sum_{j\neq i}\mathbf{m}_{ij}.\end{align*}

Finally, the node features are updated by another learnable function $\phi _{h}$: 


(5) \begin{align*}& \mathbf{h}_{i}^{l+1}=\phi_{h}\left(\mathbf{h}_{i}^{l},\mathbf{m}_{i}\right).\end{align*}

After $T$ layers of EGNN, the final node embeddings $\mathbf{F}_{\mathrm{struct}}=\left [\mathbf{h}_{1}^{T},\mathbf{h}_{2}^{T},\ldots ,\mathbf{h}_{L}^{T}\right ]\in \mathbb{R}^{L\times d_{h}}$ are obtained, where $d_{h}$ is the dimension of the hidden node embeddings.

#### Function representation

Proteins are often described using textual annotations that capture their function, which is implicitly associated with active sites of the protein. We leverage a BLM to encode these descriptions into the feature embeddings. Given a text description $\mathbf{T}=\left \{t_{1},t_{2},\ldots ,t_{n}\right \}$, where $n$ is the number of tokens, the BLM generates contextualized embeddings. To align the text representation with the sequence and structure representations, we perform global average pooling (GAP): 


(6) \begin{align*}& \mathbf{F}_{\mathrm{text}}=\mathrm{GAP}(\mathrm{BLM}(\mathbf{T}))\in\mathbb{R}^{d_{t}},\end{align*}

where $d_{t}$ is the text dimension.

### Function informed cross-attention

To effectively integrate three protein representations, we design the Function Informed Cross-attention (FunICross) module (see Supplementary Fig. S1) which consists of two symmetric branches. In the first branch, the structure modality $\mathbf{F}_{\mathrm{struct}}$ acts as the query, whereas the sequence $\mathbf{F}_{\mathrm{seq}}$ serves as key and value for the cross-attention mechanism. The attention output is added to the original query via a residual connection and layer normalization, resulting in $\mathbf{Z}_{\mathrm{struct}}$: 


(7) \begin{align*}& \mathbf{Z}_{\mathrm{struct}}=\mathrm{Norm}\left(\mathbf{Q}_{\mathrm{struct}}+\mathrm{softmax}\left( \frac{\mathbf{Q}_{\mathrm{struct}}\mathbf{K}_{\mathrm{seq}}^\top}{\sqrt{d_{s}}}\right) \mathbf{V}_{\mathrm{seq}}\right),\end{align*}

where $\mathbf{Q}_{\mathrm{struct}}=\mathbf{F}_{\mathrm{struct}}$, $\mathbf{K}_{\mathrm{seq}}=\mathbf{F}_{\mathrm{seq}}W_{\mathrm{seq}}^{K}$, $\mathbf{V}_{\mathrm{seq}}=\mathbf{F}_{\mathrm{seq}}W_{\mathrm{seq}}^{V}$. Here, $W_{\mathrm{seq}}^{K},W_{\mathrm{seq}}^{V}\in \mathbb{R}^{d_{s}\times d_{h}}$ are learnable projection matrices. Next, $\mathbf{Z}_{\mathrm{struct}}$ is concatenated with the textual feature $\mathbf{F}_{\mathrm{text}}$ (expanded to $\mathbb{R}^{d_{t}\times d_{h}}$), and passed through a feed-forward network (FFN), and finally again wrapped in residual and normalization to produce the integrated feature: 


(8) \begin{align*}& \mathbf{O}_{\mathrm{struct}}=\mathrm{Norm}\left(\mathbf{Z}_{\mathrm{struct}}+\mathrm{FFN}\left(\mathrm{Concat}\left(\mathbf{Z}_{\mathrm{struct}},\mathbf{F}_{\mathrm{text}}\right)\right)\right).\end{align*}

The second branch mirrors this process, with switching the role of structure feature and sequence feature, resulting in $\mathbf{O}_{\mathrm{seq}}$. This dual-branch design enables bidirectional integration and captures intricate cross-modal dependencies among sequence, structure, and functional information.

### Adaptive weighted fusion

To further integrate multimodal knowledge captured by FunICross with the original structure and sequence features, we proposed the Adaptive Weighted Fusion (AWF) mechanism. First, we concatenate the output of FunICross and the original structure and sequence features, resulting in $\mathbf{F}_{\mathrm{fused}}\in \mathbb{R}^{L\times \left (d_{s}+d_{h}\right )}$ and $\mathbf{F}_{\mathrm{orig}}\in \mathbb{R}^{L\times \left (d_{s}+d_{h}\right )}$, respectively. To adaptively combine both of them, we introduce a learnable weighting mechanism. Specifically, we compute a dynamic weighting coefficient $\omega ^\ast $ by: 


(9) \begin{align*}& \omega^\ast=\sigma\left(\mathrm{MLP}\left(\mathrm{Concat}\left(\mathbf{F}_{\mathrm{fused}},\mathbf{F}_{\mathrm{orig}}\right)\right)\right),\end{align*}

where $\omega ^\ast \in \left (0,1\right )$ and $\sigma (\cdot )$ represents the Sigmoid function. The final feature representation is the weighted sum of $\mathbf{F}_{\mathrm{fused}}$ and $\mathbf{F}_{\mathrm{orig}}$: 


(10) \begin{align*}& \mathbf{F}_{\mathrm{final}}=\omega^\ast\odot\mathbf{F}_{\mathrm{fused}}+\left(1-\omega^\ast\right)\odot\mathbf{F}_{\mathrm{orig}},\end{align*}

where $\odot $ denotes element-wise multiplication. This adaptive fusion mechanism ensures that the model can selectively emphasize the contributions based on their importance for the active site prediction, enabling a more flexible and robust integration of multimodal information.

### Center loss and inter loss


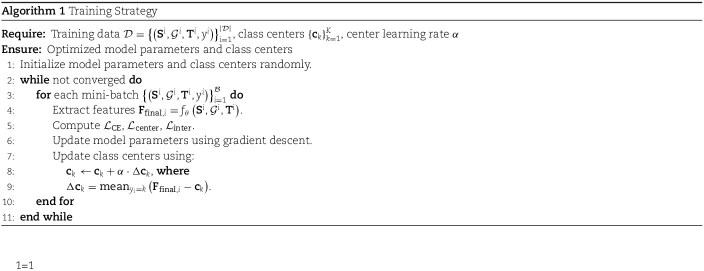


As shown in Table 1, there is a significant imbalance in the distribution of category counts. To optimize the performance of our model for imbalanced multiclass classification, we design a compound loss function that incorporates both intra-class compactness and inter-class separability, via center loss [32, 33] and inter loss, respectively.

The center loss encourages features of samples belonging to the same class to be close to their respective class centers. The loss is defined as: 


(11) \begin{align*}& \mathcal{L}_{\mathrm{center}}=\frac{1}{2\mathcal{B}}\sum_{i}^{\mathcal{B}}\Vert \mathbf{F}_{\mathrm{final},i}-\mathbf{c}_{i}\Vert^{2},\end{align*}

where $\mathcal{B}$ is the batch size, $\mathbf{F}_{\mathrm{final},i}\in \mathbb{R}^{d_{s}+d_{h}}$ and $\mathbf{c}_{i}\in \mathbb{R}^{d_{s}+d_{h}}$ are the feature of the $i$th sample and the center for class $i$ in the feature space. The class centers $\mathbf{c}_{k}$ are updated iteratively during training by: 


(12) \begin{align*}& \mathbf{c}_{k}\gets\mathbf{c}_{k}+\alpha\cdot\Delta\mathbf{c}_{k},\end{align*}

where $\Delta \mathbf{c}_{k}$ represents the mean offset of the features belonging to class $k$, and $\alpha $ is the learning rate for the centers.

The inter loss ensures that the distance between class centers remains above a predefined margin: 


(13) \begin{align*}& \mathcal{L}_{\mathrm{inter}}=\sum_{i\neq j}\max(0,m-\Vert\mathbf{c}_{i}-\mathbf{c}_{j}\Vert),\end{align*}

where $m$ is the margin that specifies the minimum allowable distance between class centers. This loss penalizes class centers that are too close, increasing inter-class separability. The final loss function is weighted combination of Cross Entropy, center loss, and inter loss: 


(14) \begin{align*}& \mathcal{L}_{\mathrm{total}}=\mathcal{L}_{\mathrm{CE}}+\lambda_{1}\mathcal{L}_{\mathrm{center}}+\lambda_{2}\mathcal{L}_{\mathrm{inter}},\end{align*}

where $\mathcal{L}_{\mathrm{CE}}$ is the cross entropy loss, $\lambda _{1}$ and $\lambda _{2}$ are balancing hyperparameters. The training process is summarized in Algorithm 1.

### Implementation details

Our implementation is based on PyTorch version 2.2.2, and all experiments were conducted on NVIDIA GeForce RTX 4090 GPUs. The evaluation metrics used include Precision, Recall, F1, AUROC, AUPRC, and MCC (detailed explanations are provided in Supplementary Note S2). For fair comparisons with other baselines, we appended additional Transformer layers and a classification head to the output of each pretrained model, ensuring a comparable scale of trainable parameters to M$^{3}$Site.

The EGNN network consists of two layers. The maximum length for protein sequences was set to 1024. Hyperparameters $\alpha $, $m$, $\lambda _{1}$, and $\lambda _{2}$ were set to 0.5, 0.1, 0.1, and 0.001, respectively. Within the CE loss, the weights assigned to the seven categories in Table 1 are 1.0, 0.7, 1.0, 0.5, 2.5, 2.0, and 0.01, respectively. The model was trained for 100 epochs using the Adam optimizer with a learning rate of 5e-5. A warm-up phase equivalent to 10% of the total steps was employed, followed by a cosine annealing scheduler for the remaining steps.

## Results

### Performance comparison with baselines

As shown in Table 2, we selected several state-of-the-art (SOTA) protein representation learning models and their variants as baselines for comparison, where the clustering threshold is 10% (we also conducted experiments at different thresholds shown as Supplementary Table S4 and Fig. S2). The baseline models include ESM-1b [34], ESM-1v [35], ESM-2-650M [36], ESM-3 [37], ProtTrans (ProtT5, ProtBert, ProtAlbert, ProtXLNet, and ProtElectra) [10], PETA [38], S-PLM [39], TAPE [40], MIF [41], and PST [42]. In addition, we also include other conventional methods [5] and a pattern-matching approach [6] for comparison in Supplementary Table S5. Some of these models utilize only protein sequences as input, while others also incorporate structural information. In our M$^{3}$Site framework, the PLM used is ESM-3, while the BLM is PubMedBERT-abs [43]. As emphasized in MMSite, it is more reasonable to draw comparisons at lower clustering thresholds, because this setting better reflects real-world scenarios where models are required to generalize to unseen and diverse sequences, thereby providing a more robust evaluation. The loss function plots and F1 score learning curve are shown in Supplementary Fig. S3. As indicated in Table 2, M$^{3}$Site significantly outperforms all other models across seven evaluation metrics, demonstrating its strong capability in accurately identifying and classifying protein active sites.

**Table 2:** Performance comparison of M$^{3}$Site and other SOTA baselines on multiclass active site identification, using the dataset clustered at a 10% similarity threshold

		Modality input								
Model	Version	Sequence	Structure	Function	Precision	Recall	F1	AUROC	AUPRC	MCC
ESM	1b	Yes	No	No	0.4709	0.4730	0.4719	0.6760	0.5091	0.0000
	1v	Yes	No	No	0.4728	0.4748	0.4738	0.5092	0.4848	0.0000
	2-650M	Yes	No	No	0.4650	0.4669	0.4660	0.6080	0.4914	0.0001
	3	Yes	No	No	0.4747	0.4768	0.4757	0.5699	0.4970	0.0000
ProtT5	BFD	Yes	No	No	0.4957	0.4976	0.4966	0.6769	0.5719	0.0417
	UniRef	Yes	No	No	0.4917	0.4937	0.4927	0.7049	0.5732	0.0339
ProtBert	BFD	Yes	No	No	0.4745	0.4766	0.4755	0.4485	0.4856	0.0000
	UniRef	Yes	No	No	0.4747	0.4768	0.4757	0.4649	0.4832	−0.0002
ProtAlbert		Yes	No	No	0.4198	0.4201	0.4199	0.5938	0.4900	−0.0033
ProtXLNet		Yes	No	No	0.4745	0.4766	0.4755	0.6444	0.5161	0.0000
ProtElectra		Yes	No	No	0.4739	0.4759	0.4749	0.4178	0.4973	0.0023
PETA	deep_base	Yes	No	No	0.4744	0.4765	0.4754	0.5472	0.5045	0.0001
S-PLM		Yes	No	No	0.4720	0.4743	0.4731	0.3607	0.4781	0.0000
TAPE		Yes	No	No	0.4739	0.4759	0.4749	0.5343	0.4970	0.0000
MIF	MIF	Yes	Yes	No	0.4743	0.4763	0.4753	0.6211	0.5051	0.0000
	MIF-ST	Yes	Yes	No	0.4741	0.4761	0.4751	0.3846	0.4834	0.0000
PST	t33	Yes	Yes	No	0.4726	0.4746	0.4736	0.4155	0.4804	0.0001
	t33_so	Yes	Yes	No	0.4741	0.4765	0.4752	0.4215	0.4800	0.0007
**M$^{\bf 3}$Site**	**3-abs**	**Yes**	**Yes**	**Yes**	**0.8041**	**0.8373**	**0.8133**	**0.9831**	**0.8949**	**0.6562**

Note: The best evaluation values are shown in bold.

### Performance comparison of protein language model and biomedical language model variants

To assess the impact of different pretrained models on M$^{3}$Site’s performance, we conducted a systematic evaluation by replacing PLM and BLM with various SOTA alternatives. Specifically, we tested the performance using multiple PLMs, including ProtT5-BFD, ProtT5-UniRef, ESM-1b, ESM-2-650M, and ESM-3, and experimented with two versions of PubMedBERT (full and abs). These models were chosen because they represent widely recognized pretrained language models. Table 3 summarizes the performance of M$^{3}$Site under these configurations. For the PLM, we observed that ESM-3 consistently outperformed other variants, due to its enhanced architecture and improved representation learning of protein sequences. For the BLM, PubMedBERT-abs achieved the best results within most metrics.

**Table 3:** Performance comparison of PLM and BLM variants in M$^{3}$Site

PLM	BLM	Precision	Recall	F1	AUROC	AUPRC	MCC
ProtT5-BFD	PubMedBERT-full	0.7761	0.8069	0.7852	0.9176	0.8612	0.6177
ProtT5-BFD	PubMedBERT-abs	0.7890	0.8136	0.7968	0.9397	0.8722	0.6378
ProtT5-UniRef	PubMedBERT-full	0.7630	0.8014	0.7742	0.9396	0.8664	0.6146
ProtT5-UniRef	PubMedBERT-abs	0.7730	0.7953	0.7792	0.9374	0.8557	0.6061
ESM-1b	PubMedBERT-full	0.7707	0.7709	0.7725	0.8943	0.8467	0.5823
ESM-1b	PubMedBERT-abs	0.7768	0.7842	0.7776	0.8774	0.8414	0.5826
ESM-2-650M	PubMedBERT-full	0.7817	0.7932	0.7853	0.9330	0.8696	0.5881
ESM-2-650M	PubMedBERT-abs	0.7832	0.7939	0.7861	0.9194	0.8608	0.6018
ESM-3	PubMedBERT-full	0.8037	0.8341	0.8121	0.9828	**0.8950**	0.6532
ESM-3	PubMedBERT-abs	**0.8041**	**0.8373**	**0.8133**	**0.9831**	0.8949	**0.6562**

Note: The best evaluation values are shown in bold.

**Figure 2:**
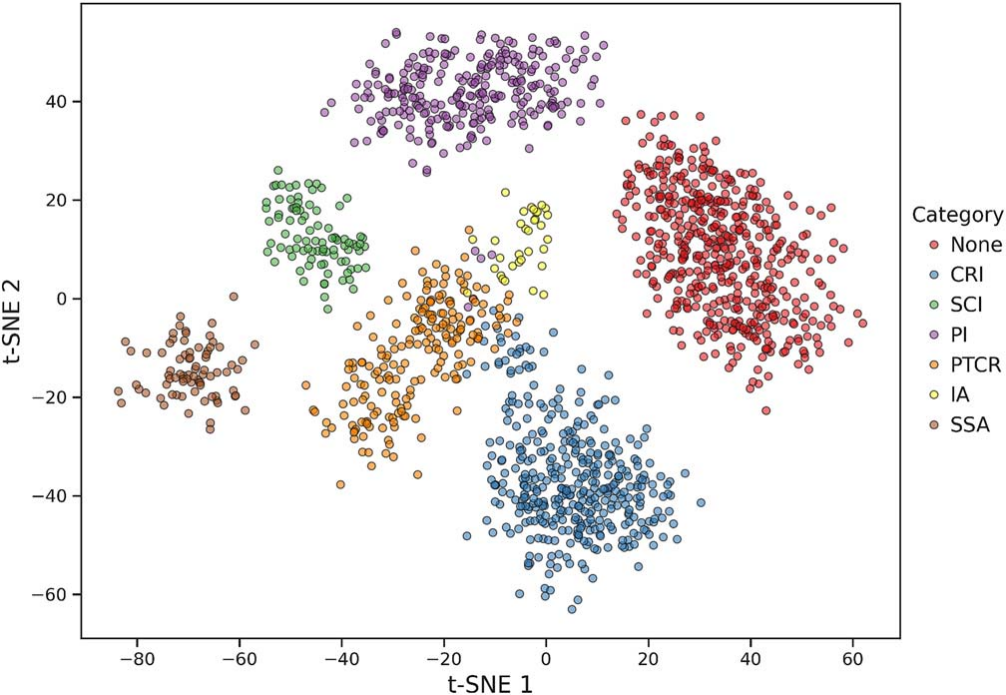
t-SNE visualization of feature representations for multiclass active site identification, where M$^{3}$Site-ESM3-abs is used.

To further analyze M$^{3}$Site’s ability to distinguish among classes, we used t-SNE [44] to project the multimodal output representations into a 2D space, as shown in Fig. 2. This visualization provides insights into the distribution of samples across different categories in the feature space. It is evident that most classes are well separated, demonstrating the model’s discriminative ability for multiclass active site classification. Additionally, to highlight the interpretability of intermediate output, we further visualized the per-residue output confidence scores of two FunICross branches and the fusion result in Supplementary Fig. S4. The comparison indicates M$^{3}$Site’s ability to integrate complementary information and achieve a more accurate identification, demonstrating the effectiveness of our multimodal strategy.

### Ablation studies

To validate the effectiveness of individual components and loss terms, we conducted ablation studies, with results summarized in Table 4. Removing any of the three modalities (sequence, structure, or function) led to a significant performance degradation, with F1 scores decreasing by 37.70%, 10.66%, and 6.05%, respectively, when sequence, structure, or function is removed. Excluding the FunICross module and initial pretrained features $\mathbf{F}_{\mathrm{orig}}$ also led to declines, underscoring their contributions to the model’s performance. Replacing the AWF mechanism with a simple addition operation also reduced performance, demonstrating AWF’s role in adaptively balancing multimodal information. In the loss ablation study, removing the $\mathcal{L}_{\mathrm{inter}}$ or $\mathcal{L}_{\mathrm{center}}$ resulted in moderate declines, while removing both caused the largest performance drop, illustrating the complementary characteristic of these two terms. Additionally, we performed a leave-one-class-out evaluation of M$^{3}$Site’s class-discriminative capacity in Supplementary Table S6 to confirm our model captures class-specific signatures rather than generic “active site” patterns.

**Table 4:** Ablation study results for each component and loss term in M$^{3}$Site-ESM3-abs

Model	Precision	Recall	F1	AUROC	AUPRC	MCC
**M$^{\bf 3}$Site-ESM3-abs**	**0.8041**	**0.8373**	**0.8133**	**0.9831**	**0.8949**	**0.6562**
*Component ablation study*						
*w/o* sequence input	0.5053	0.5088	0.5067	0.5853	0.5358	0.0396
*w/o* structure input	0.6933	0.7708	0.7266	0.9275	0.8504	0.5582
*w/o* function input	0.7600	0.7746	0.7641	0.9205	0.8488	0.5717
*w/o* FunICross	0.7530	0.8348	0.7793	0.9785	0.8868	0.6216
*w/o* $\mathbf{F}_{\mathrm{orig}}$ feature	0.7751	0.7908	0.7797	0.9097	0.8610	0.5854
AWF $\rightarrow $ addition	0.7769	0.8372	0.7946	0.9799	0.8801	0.6270
*Loss ablation study*						
*w/o* $\mathcal{L}_{\mathrm{inter}}$	0.7886	0.8317	0.8034	0.9830	0.8911	0.6453
*w/o* $\mathcal{L}_{\mathrm{center}}$	0.7674	0.8315	0.7860	0.9814	0.8770	0.6125
*w/o* $\mathcal{L}_{\mathrm{inter}}$, $\mathcal{L}_{\mathrm{center}}$	0.7638	0.8294	0.7829	0.9819	0.8774	0.6097

Note: The best evaluation values are shown in bold.

### Case study and model deployment

**Figure 3:**
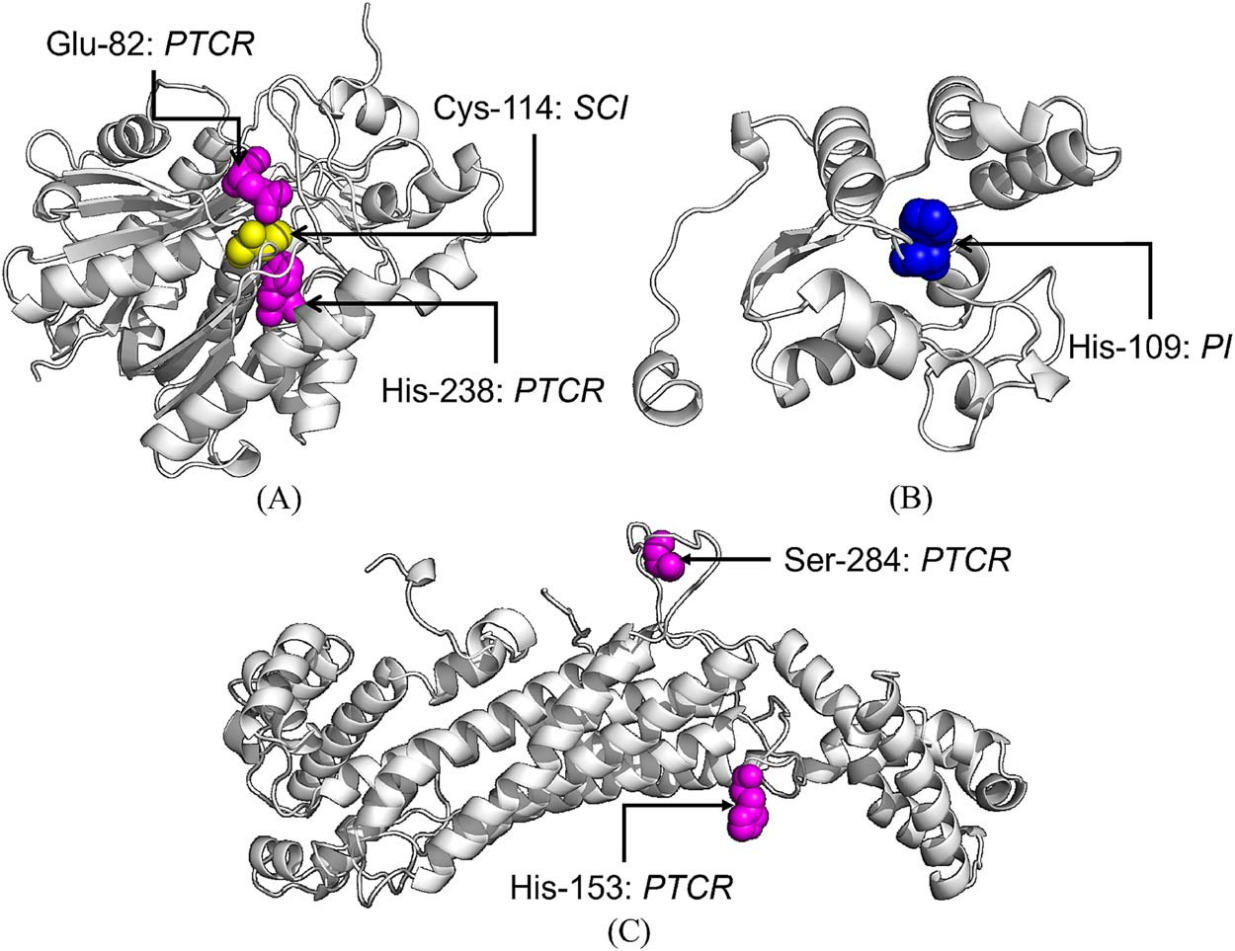
Visualization of multiclass active site identification results of proteins by M$^{3}$Site-ESM3-abs where the UniProt IDs of proteins (A), (B), and (C) are A0A384E143, A0A3Q0KJ78, and G4VQX9, with lengths of 349, 143, and 480, respectively.

To further evaluate the generalizability, we conducted a case study using unseen data collected from UniProt after 28 July 2024, while training and validation data were from before this date. Following the standards outlined in Section “Benchmark dataset”, we identified 33 samples for evaluation. Impressively, M$^{3}$Site achieved a overall Precision of 0.899, a Recall of 0.949, an F1 of 0.912, an AUROC of 0.999, an AUPRC of 0.966, and an MCC of 0.837 on these unseen samples. Taking proteins with UniProt IDs A0A384E143, A0A3Q0KJ78, and G4VQX9 as examples, M$^{3}$Site successfully identified 1–3 active site categories among hundreds of amino acids with near-perfect performance. We visualized the identification results as shown in Fig. 3, and evaluated the average inference time (“preprocessing” refers to the process of generating embeddings) of M$^{3}$Site and other two baselines on different hardware configurations in Supplementary Table S7.

Furthermore, to create a stringent family-level out-of-distribution evaluation, we held out all Glycosyl-Hydrolase (GH) families listed in Pfam as the test set, and the remaining non-GH families formed the training set. We guarantee that no family name, Pfam clan, SCOPe/CATH superfamily, or sequence with >10% identity appears in both splits. Our M$^{3}$Site still achieves high performance (Precision of 0.829, Recall of 0.995, F1 of 0.884, AUROC of 0.999, and MCC of 0.800) in this scenario.

In addition, we developed an interactive application based on Gradio, enabling users to easily predict, visualize, and analyze active sites in proteins (see Supplementary Note S3). By bridging complex models with accessible visualization and analysis, this application enhances the practical utility of M$^{3}$Site, making it a valuable tool for experimental and computational researchers.

## Discussion

Our experimental results demonstrate the advantages of integrating protein sequence, structural, and functional text modalities for active site prediction. Ablation studies reveal that each modality provides unique and complementary information; notably, the removal of structural or functional text inputs led to substantial performance degradation, with the sequence modality serving as the most foundational component. This confirms that spatial context derived from structure and semantic guidance from functional text are crucial for accurate identification. The proposed FunICross module and AWF mechanism were also critical, as their removal or simplification resulted in marked performance declines, confirming their role in enabling adaptive and informative cross-modal interactions. Moreover, M^3^Site substantially outperforms a wide range of SOTA baselines across all metrics, particularly under the 10% threshold. This underscores its superior generalization capability to evolutionarily distant proteins. The model's robustness is further evidenced by high performance on temporally separated and family-level out-of-distribution test sets.

Despite these strengths, M^3^Site still has certain limitations. It depends on high-confidence 3D structural data, and its functional categories are derived from LLM-assisted clustering and expert refinement. Although this approach ensures biological relevance, it may overlook fine-grained functional variations. Nevertheless, our framework establishes a scalable foundation. Importantly, the multimodal design can naturally be extended or fine-tuned to accommodate more specific classifications as additional data becomes available. This flexibility facilitates future exploration of detailed functional specificity.

## Conclusion

In this study, we proposed M$^{3}$Site, a robust multimodal framework for residue-level protein active site classification. By integrating sequence embeddings from PLM, structural representations from EGNN, and functional annotations from BLM, M$^{3}$Site achieves SOTA performance across multiple evaluation metrics. Our FunICross module efficiently fuses multimodal information through symmetric cross-attention, while the AWF module balances the contributions of each modality. Additionally, the compound loss function enhances model robustness against dataset imbalance. Experimental results demonstrate the effectiveness of M$^{3}$Site compared to existing baselines, and our interactive interface facilitates practical use in biological research. Future work may explore extending M$^{3}$Site to predict more granular functional categories and generalize across diverse protein families.

**Key Points**

- We construct a high-quality dataset of 25 883 proteins with residue-level functional annotations refined by large language model and human experts.
- We propose M$^{3}$Site, a multimodal framework that identifies active sites and classifies their functional roles, combining protein sequences, structure, and functional text descriptions.
- Comprehensive experiments show M$^{3}$Site’s state-of-the-art effectiveness and practical utility in protein active site analysis.
- We develop an interface for interactive prediction, visualization, and analysis of protein active sites.

## Supplementary Material

supplementary_bbaf590

## Data Availability

The dataset, source code for experiments, and interactive application associated with this study are publicly available at https://github.com/Gift-OYS/M3Site.
