# Supplementary material for: M3Site: multiclass multimodal learning for protein active site identification and classification
Source: Brief Bioinform. 2025 Nov 12;26(6):bbaf590. doi: 10.1093/bib/bbaf590 (PMC12609176; doi:10.1093/bib/bbaf590)
Supplement: supplementary_bbaf590 [file supplementary_bbaf590.docx]

**Supplementary Tables**

**Supplementary Table S1.** Re-clustered active site categories of active sites in UniProt

| **No.** | **Raw Annotations in UniProt** | **Categories** | **Descriptions** |
| --- | --- | --- | --- |
| 1 | None | Not an active site | Not an active site |
| 2 | Schiff-base intermediate with acetaldehyde | Covalent Reaction Intermediates | Active sites forming covalent reaction intermediates, typically through imine (Schiff-base) or related linkages with substrates during catalysis |
| 3 | Schiff-base intermediate with DNA |  |  |
| 4 | Schiff-base intermediate with DXP |  |  |
| 5 | Schiff-base intermediate with substrate |  |  |
| 6 | for decarboxylase activity |  |  |
| 7 | S-methylcysteine intermediate | Sulfur-containing Covalent Intermediates | Active sites forming covalent intermediates via sulfur-containing residues |
| 8 | Cysteine persulfide intermediate |  |  |
| 9 | Cysteine sulfenic acid (-SOH) intermediate |  |  |
| 10 | S-selanylcysteine intermediate |  |  |
| 11 | Cysteine radical intermediate |  |  |
| 12 | Thioimidate intermediate |  |  |
| 13 | Thioimide intermediate |  |  |
| 14 | Acyl-thioester intermediate |  |  |
| 15 | Glycyl thioester intermediate |  |  |
| 16 | Pros-phosphohistidine intermediate | Phosphorylated Intermediates | Active sites forming covalent phosphorylated intermediates on residues such as histidine, serine, or threonine |
| 17 | Tele-phosphohistidine intermediate |  |  |
| 18 | Phosphoserine intermediate |  |  |
| 19 | Proton donor | Proton Transfer & Charge Relay Systems | Active sites involved in proton transfer and charge relay mechanisms |
| 20 | Proton donor/acceptor |  |  |
| 21 | Charge relay |  |  |
| 22 | Involved in ionization of N3 of dUMP, leading to its activation |  |  |
| 23 | For Fru-6P isomerization activity | Isomerization Activity | Active sites catalyzing structural rearrangement of molecules without changing molecular formula |
| 24 | Specific for D-alanine | Substrate-specific Activities | Active sites conferring specificity for a particular substrate |
| 25 | Specific for L-alanine |  |  |

**Supplementary Table S2.** Statistics of dataset split at different sequence similarity thresholds

| **Dataset** | **10%** | **30%** | **50%** | **70%** | **90%** |
| --- | --- | --- | --- | --- | --- |
| Train | 5613 | 6255 | 11724 | 17848 | 19988 |
| Validation | 726 | 805 | 1385 | 2213 | 2464 |
| Test | 707 | 766 | 1483 | 2230 | 2428 |

**Supplementary Table S3.** Composition of 62-dimensional node features in protein structure representation.

| **Feature Type** | **Dimension** | **Description** |
| --- | --- | --- |
| Secondary Structure | 9 | One-hot encoding of 8 DSSP secondary structure types (H, B, E, G, I, T, S, -), with an additional "unknown" type. |
| Dihedral Angles | 16 |  |
| - $\phi$ angle | 2 | Sine and cosine of the $\phi$ angle. |
| - $\psi$ angle | 2 | Sine and cosine of the $\psi$ angle. |
| - $\omega$ angle | 2 | Sine and cosine of the $\omega$ angle. |
| - $\chi_{1}$to $\chi_{5}$ angles | 10 | Sine and cosine of $\chi_{1}$to $\chi_{5}$ side-chain torsion angles, calculated based on residue-specific atoms. |
| Atomic Features | 7 |  |
| - Atomic mass | 1 | Atomic mass of each residue's atom. |
| - B-factor | 1 | Temperature factor for atomic motion. |
| - Side-chain status | 1 | Binary value indicating whether the atom is part of the residue's side-chain. |
| - Electronic charge | 1 | Formal electronic charge of the atom. |
| - Bonded hydrogens | 1 | Number of hydrogens bonded to the atom. |
| - Ring membership | 1 | Binary value indicating whether the atom is part of a ring structure. |
| - Van der Waals radius | 1 | Van der Waals radius of the atom. |
| Hydrogen Bond Features | 2 | Mean hydrogen bond properties calculated using MDtraj. |
| Pseudo Position Embedding | 1 | Scaled Euclidean distance from a reference centroid to other residues. |
| Residue Features | 27 |  |
| - Amino acid type | 21 | One-hot encoding of the residue type (20 standard amino acids + 1 for “unknown”). |
| - Hydrophobicity | 1 | Hydrophobicity value of the amino acid. |
| - Polarity | 1 | Binary indicator for whether the amino acid is polar. |
| - Charge | 1 | Net charge of the amino acid. |
| - pKa | 1 | pKa value of the amino acid. |
| - Volume | 1 | Molecular volume of the amino acid. |
| - Mass | 1 | Molecular mass of the amino acid. |

**Supplementary Table S4.**Performance comparison of M^3^Site and other SOTA baselines on multi-class active site identification, using the dataset clustered at a 30% similarity threshold.

| **Model** | **Version** | **Modality Input** | | | **Precision** | **Recall** | **F1** | **AUROC** | **AUPRC** | **MCC** |
| --- | --- | --- | --- | --- | --- | --- | --- | --- | --- | --- |
|  |  | **Seq.** | **Struct.** | **Fun.** |  |  |  |  |  |  |
| ESM | 1b | Yes | No | No | 0.4867 | 0.4885 | 0.4876 | 0.5029 | 0.4936 | 0.0000 |
|  | 1v | Yes | No | No | 0.4731 | 0.4761 | 0.4751 | 0.6612 | 0.5223 | 0.0000 |
|  | 2-650M | Yes | No | No | 0.4869 | 0.4887 | 0.4878 | 0.6285 | 0.4997 | 0.0000 |
|  | 3 | Yes | No | No | 0.4763 | 0.4783 | 0.4773 | 0.5433 | 0.4957 | 0.0000 |
| ProtT5 | BFD | Yes | No | No | 0.5734 | 0.5739 | 0.5735 | 0.8312 | 0.7102 | 0.1918 |
|  | UniRef | Yes | No | No | 0.5621 | 0.5631 | 0.5625 | 0.8163 | 0.6528 | 0.2020 |
| ProtBert | BFD | Yes | No | No | 0.4808 | 0.4828 | 0.4818 | 0.5370 | 0.4957 | 0.0000 |
|  | UniRef | Yes | No | No | 0.4749 | 0.4769 | 0.4759 | 0.4375 | 0.4829 | 0.0001 |
| ProtAlbert |  | Yes | No | No | 0.4350 | 0.4365 | 0.4358 | 0.5994 | 0.5051 | 0.0006 |
| ProtXLNet |  | Yes | No | No | 0.4761 | 0.4781 | 0.4771 | 0.6340 | 0.5446 | 0.0000 |
| ProtElectra |  | Yes | No | No | 0.4692 | 0.4711 | 0.4702 | 0.3885 | 0.4948 | 0.0004 |
| PETA | deep_base | Yes | No | No | 0.4759 | 0.4779 | 0.4769 | 0.5065 | 0.5092 | 0.0000 |
| S-PLM |  | Yes | No | No | 0.4529 | 0.4547 | 0.4538 | 0.3023 | 0.4789 | 0.0001 |
| TAPE |  | Yes | No | No | 0.4743 | 0.4763 | 0.4753 | 0.5701 | 0.5079 | 0.0000 |
| MIF | MIF | Yes | Yes | No | 0.4759 | 0.4779 | 0.4769 | 0.6078 | 0.5088 | 0.0000 |
|  | MIF-ST | Yes | Yes | No | 0.4765 | 0.4786 | 0.4775 | 0.3799 | 0.4939 | 0.0000 |
| PST | t33 | Yes | Yes | No | 0.4710 | 0.4727 | 0.4719 | 0.4272 | 0.4859 | 0.0000 |
|  | t33_so | Yes | Yes | No | 0.4731 | 0.4750 | 0.4741 | 0.4668 | 0.4850 | 0.0000 |
| M^3^Site | 3-abs | Yes | Yes | Yes | 0.8829 | 0.9061 | 0.8882 | 0.9773 | 0.9473 | 0.8083 |

**Supplementary Table S5.**Performance comparison of M^3^Site-ESM3-abs and other classical baselines, including Random Forest, Support Vector Machine and Discern, on multi-class active site identification, using the dataset clustered at a 10% similarity threshold.

| **Model** | **Modality Input** | | | **Precision** | **Recall** | **F1** | **AUROC** | **AUPRC** | **MCC** |
| --- | --- | --- | --- | --- | --- | --- | --- | --- | --- |
|  | **Seq.** | **Struct.** | **Fun.** |  |  |  |  |  |  |
| Random Forest | Yes | No | No | 0.7076 | 0.2428 | 0.3086 | 0.7660 | 0.3903 | 0.3817 |
| Support Vector Machine | Yes | No | No | 0.6208 | 0.2553 | 0.3211 | 0.7997 | 0.3423 | 0.3857 |
| Discern | Yes | Yes | No | 0.4045 | 0.2493 | 0.2871 | 0.8234 | 0.3361 | 0.4045 |
| M^3^Site-ESM3-abs | Yes | Yes | Yes | 0.8829 | 0.9061 | 0.8882 | 0.9773 | 0.9473 | 0.8083 |

**Supplementary Table S6.**Leave-one-class-out evaluation of M^3^Site’s class-discriminative capacity at the 10% similarity threshold. In each trial, one class is excluded from the training set. The table presents the number of residues in the withheld class, along with their counts and percentages predicted as “Non-active” or assigned to any of the remaining active-site classes.

| **Held-out Class** | **Residue Count** | **Predicted Non-active** | **Predicted Any Other Active** |
| --- | --- | --- | --- |
| CRI | 355 | 349 (98.31%) | 6 (1.69%) |
| SCI | 1108 | 1067 (96.30%) | 41 (3.70%) |
| PI | 434 | 426 (98.16%) | 8 (1.84%) |
| PTCR | 6027 | 5676 (94.18%) | 351 (5.82%) |
| IA | 16 | 16 (100.00%) | 0 (0.00%) |
| SSA | 68 | 65 (95.59%) | 3 (4.41%) |

**Supplementary Table S7.**Average inference time of M^3^Site and other two baselines on different hardware configurations. “Preprocessing” refers to the process of generating embeddings. The unit is seconds per sample. The hardware configurations include a single GPU (NVIDIA A100-PCIE-80GB or NVIDIA GeForce RTX 4090) and a CPU (AMD EPYC 7V13 or Intel Xeon Platinum 8375C).

| **Method** | **Modality Input** | **RTX A100** | **RTX 4090** | **AMD CPU** | **Intel CPU** |
| --- | --- | --- | --- | --- | --- |
| ESM-3 (Preprocessing+inference) | Seq. | 0.7156 | 1.8155 | 2.0944 | 3.9806 |
| MMSite (Preprocessing+inference) | Seq.+Fun. | 0.8605 | 1.9701 | 2.3084 | 4.2963 |
| M^3^Site (Only inference) | Seq.+Struct.+Fun. | 0.0057 | 0.0581 | 0.0403 | 2.3046 |
| M^3^Site (Preprocessing+inference) | Seq.+Struct.+Fun. | 1.1158 | 2.2200 | 2.5733 | 5.2489 |

**Supplementary Figures**


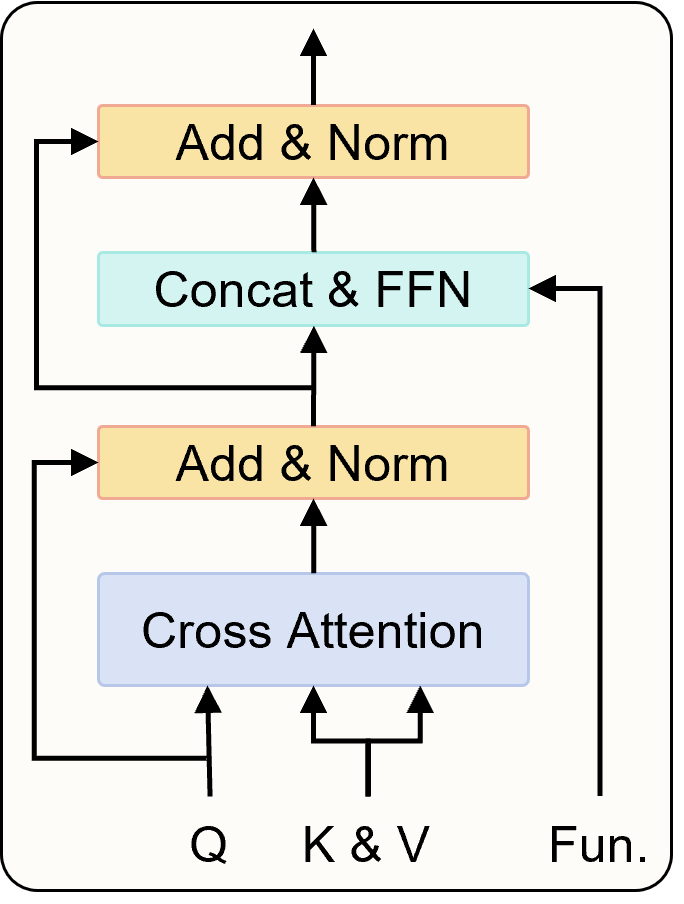


**Supplementary Figure S1. Architecture of the FunICross module.** The cross-attention operation computes interactions between modalities to extract meaningful correlations. The output of the cross-attention layer is then added to the original query via a residual connection and normalized through an Add & Norm layer. Next, the output is concatenated with the functional text features (Fun.) and passed through an FFN, which helps integrate information across all modalities. The FFN’s output is further combined with the result of the first Add & Norm step using another Add & Norm operation to finalize the fusion process.

**
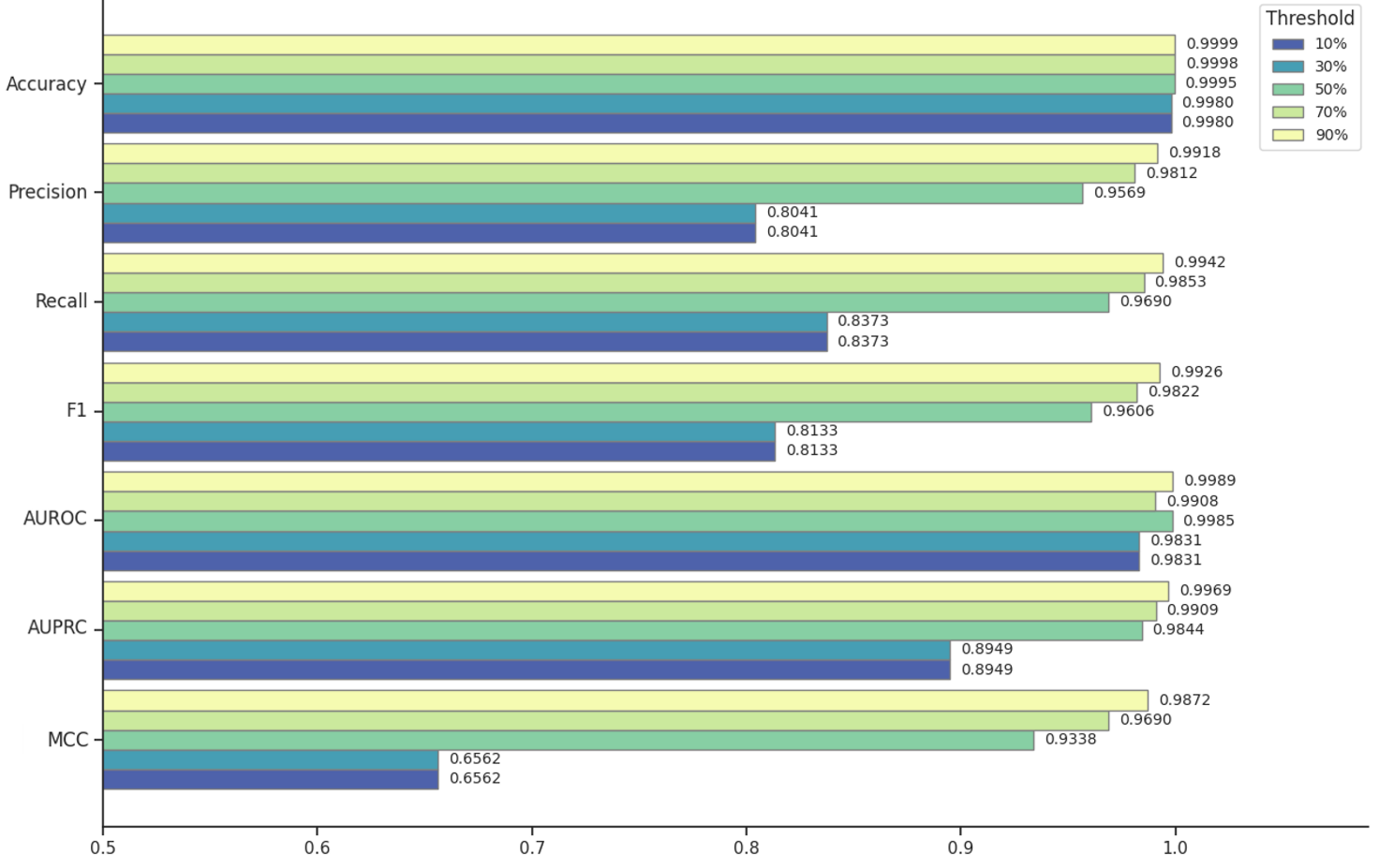
**

**Supplementary Figure S2. Performance comparison across clustering identity thresholds of 10%, 30%, 50%, 70%, and 90%.** The results show a clear trend of improved performance across all metrics as the threshold increases, due to greater similarity between training and testing data and a larger training dataset (Table S2). However, lower thresholds provide a more rigorous evaluation setting by ensuring that the test data is more distinct from the training data. Therefore, it is more reasonable to make comparisons at lower thresholds.

**
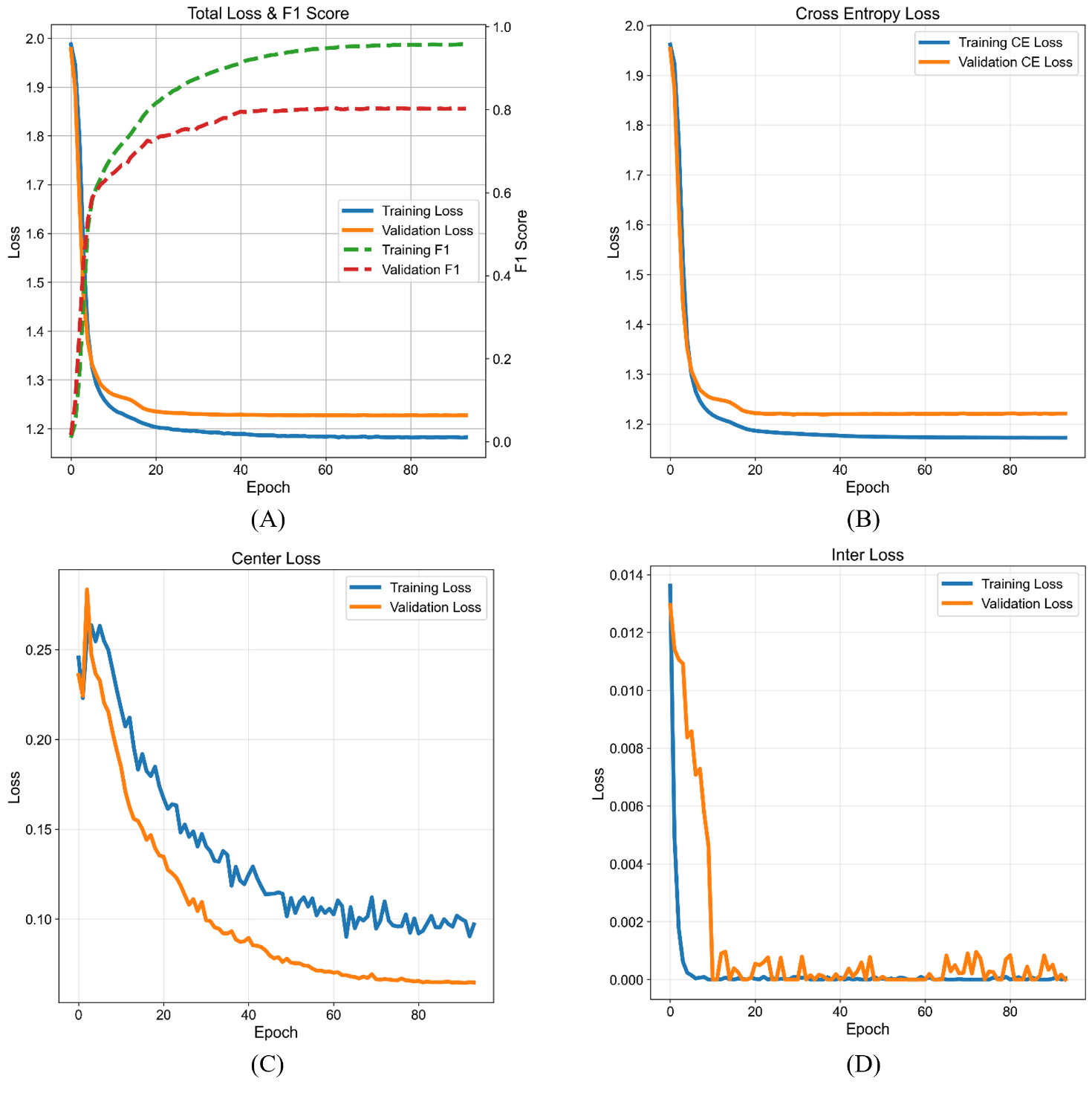
**

**Supplementary Figure S3. Loss functions and learning curves of the training process of M^3^Site under the similarity threshold of 10%.** (A) Total loss and F1 score trajectories across training/validation sets during training. (B) Cross Entropy Loss progression. (C) Center Loss progression. (D) Inter Loss progression.

**
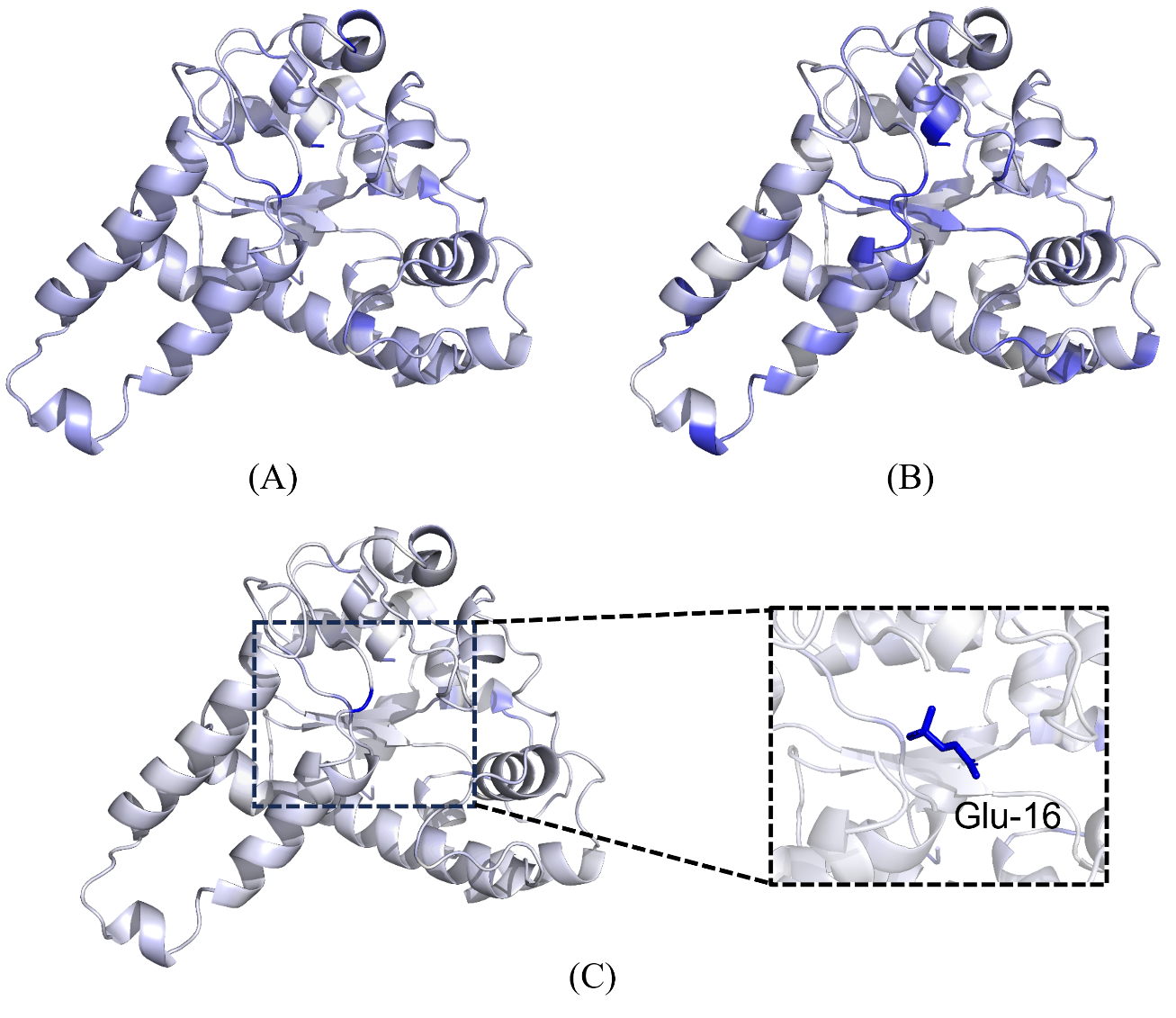
**

**Supplementary Figure S4. Visualization of predicted confidence scores for active site identification on the protein with UniProt ID: Q20351 at different stages of M^3^Site;** **darker shades denote higher confidence.** (A) Prediction confidence from the sequence branch **O**_seq_ in FunICross; (B) Prediction confidence from the structure branch **O**_struct_ in FunICross; (C) Prediction confidence after fusing both branches, where the residue Glu-16 receives the highest score and corresponds to the only annotated active site in this protein. This case illustrates the effectiveness of our multi-modal design in leveraging the strengths of each modality.

**Supplementary Note S1 (Prompt for LLM clustering)**

The prompt used for the LLM to perform initial clustering on raw functional annotations are as follows:

| **Prompt for LLM clustering** |
| --- |
| You are an expert specializing in the classification of protein active site function. Your task is to categorize the given active site function descriptions into **5 to 10 distinct categories**, and provide a detailed description of each category.  **Ensure that the categories you define are consistent, with each representing a biological or chemical function that shares similar characteristics or meanings.**  The raw descriptions of the protein active site functions are provided below:  {active_site_function_descriptions_list}  **Your response should be a detailed classification result in the following JSON format:**  ```json  CLASSIFICATION_RESULT = [  {  "category": "Category Name 1",  "active_site_function_descriptions": [  "Active site function description 1",  "Active site function description 2",  ...  ],  "description": "Provide a 2-3 sentence description of the category"  },  ...  ]  ```  In case any active site function description could potentially belong to multiple categories, please explain these uncertain descriptions in detail and suggest the categories they might belong to. This will assist human experts in making the final classification decision.  PLEASE NOTE: Descriptions that do not clearly fit into any existing category should be treated as "uncertain descriptions". **These should not appear in the above CLASSIFICATION_RESULT dictionary.** Instead, they should be provided in another JSON format with the following structure:  ```json  UNCERTAIN_DESCRIPTIONS = [  {  "active_site_function_description": "Active site function description",  "explanation": "Provide a 2-3 sentence explanation of why this description is uncertain",  "Suggested categories": ["Category Name 1", "Category Name 2"]  },  ...  ]  ``` |

**Supplementary Note S2 (Metrics Introduction)**

In our multi-class active site identification and classification task, we employed Precision, Recall, F1, AUROC, AUPRC, and MCC to assess the performance of our model. Below, we provide explanations of the meaning of them and their calculations.

**(1) Precision**

Precision evaluates the proportion of correctly predicted residues for a specific class relative to all residues that were predicted to belong to that class. For multi-class tasks, we compute the macro-Precision as:

where is the total number of classes.

**(2) Recall**

Recall measures the proportion of correctly predicted residues for a specific class relative to all actual residues belonging to that class. Like Precision, we compute the macro-Recall as:

**(3) F1**

The F1 score is the harmonic mean of Precision and Recall, providing a single measure that balances the trade-off between false positives and false negatives. We compute the macro-F1 as:

**(4) AUROC**

AUROC measures the model’s ability to discriminate between classes by computing the area under the Receiver Operating Characteristic (ROC) curve. The ROC curve plots the Recall against the False Positive Rate (FPR) at various classification thresholds.

AUROC ranges from 0 to 1, where a higher value indicates better discrimination between positive and negative classes. In our batch-based evaluation, not all classes may appear in every batch. Therefore, for binary cases, AUROC is computed directly on the positive class, while for multi-class cases, the *one-vs-one* strategy is used to calculate pairwise AUROC scores and average them.

**(5) AUPRC**

AUPRC measures the area under the Precision-Recall curve. Similar to AUROC, AUPRC is dynamically calculated based on the unique classes present in each batch. Probabilities are trimmed to match the observed classes, and true labels are converted into one-hot encoding for consistency. The macro-average Precision-Recall score is then computed to ensure a balanced evaluation across all present classes.

**(6) MCC**

MCC is a balanced metric that works well even for imbalanced datasets. For binary classification:

where TP, TN, FP, and FN represent True Positives, True Negatives, False Positives, and False Negatives, respectively. For multi-class classification, MCC is generalized by using the confusion matrix. MCC ranges from -1 to 1, where higher value indicates better predictions performance.

**Supplementary Note S3 (Usage Demo of Application)**

To facilitate broader adoption and practical usability of M^3^Site, we developed an interactive front-end application based on Gradio (with the help of gradio-molecule3d: https://pypi.org/project/gradio-molecule3d/). As shown in Supplementary Figure S5, the interface is divided into three main sections: General Settings, Prediction Panel, and Result Details. The General Settings section allows users to configure key parameters before running the M^3^Site model, such as pretrained model variants, visualization styles and colors. In Prediction Panels, users can upload a protein structure file or fetch it directly from web. Additionally, users can provide optional functional prompts to guide the model during prediction. As for Result Details, result information about each predicted site, including Active Site Type, Residue Type, Residue Number, and Confidence Score will be shown here. Supplementary Figure S6 and Supplementary Figure S7 present an example of predicted protein active site results along with an interactive visualization for analysis.


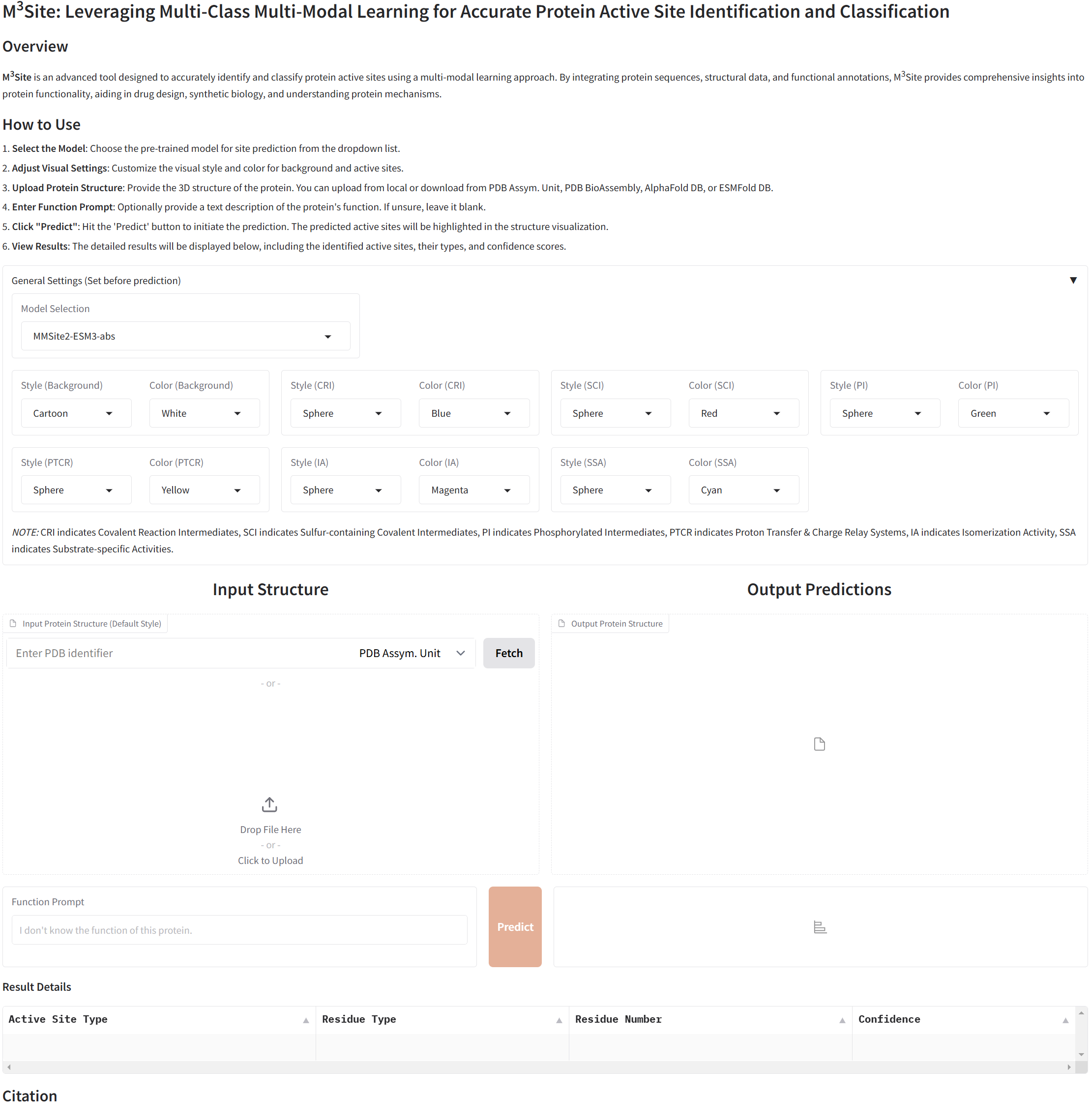


**Supplementary Figure S5. Interface overview.** It contains three sections: General Settings, Prediction Panel, and Result Details.


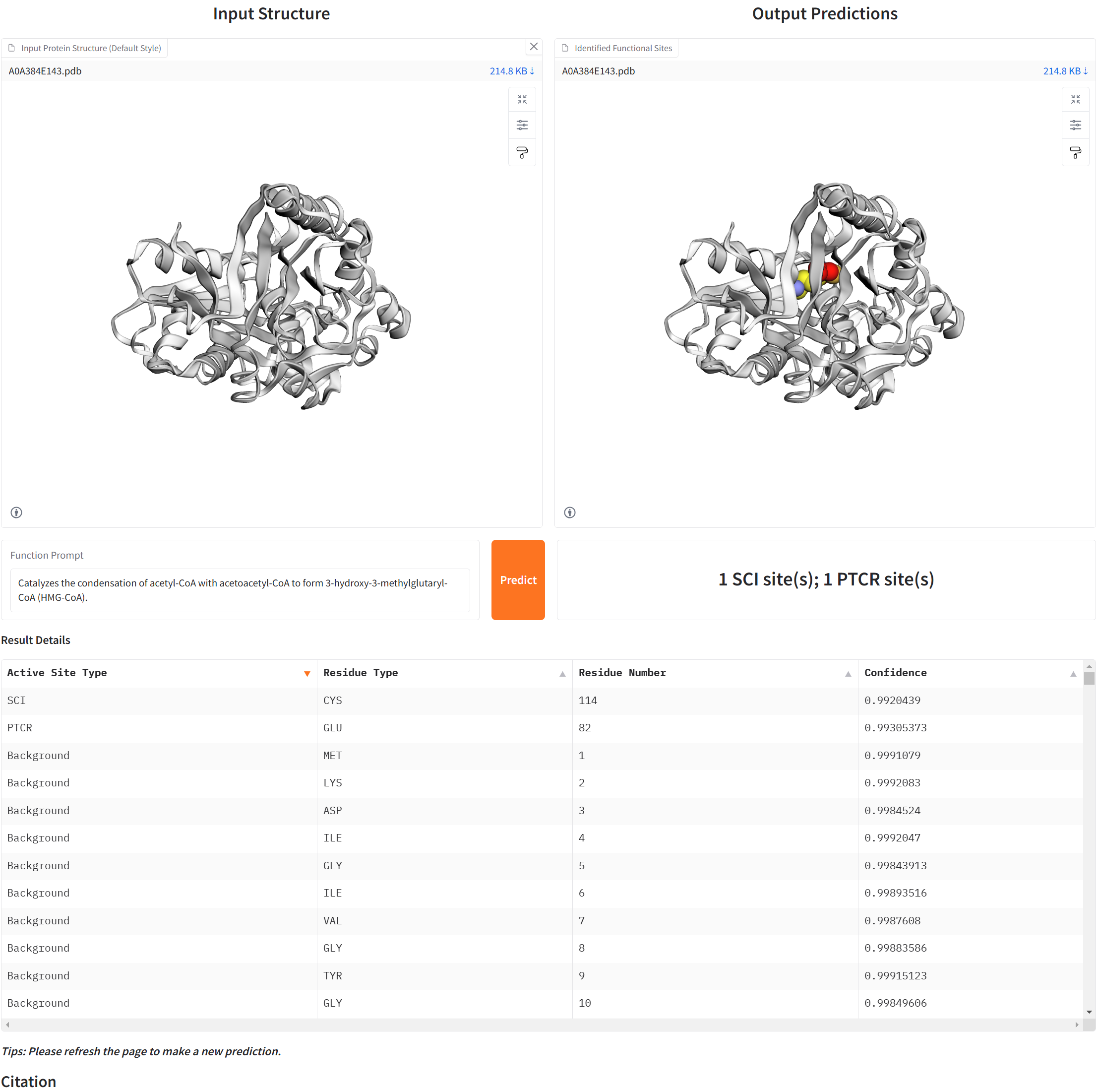


**Supplementary Figure S6. Example of predicted protein active site results using M^3^Site.** UniProt ID: A0A384E143.


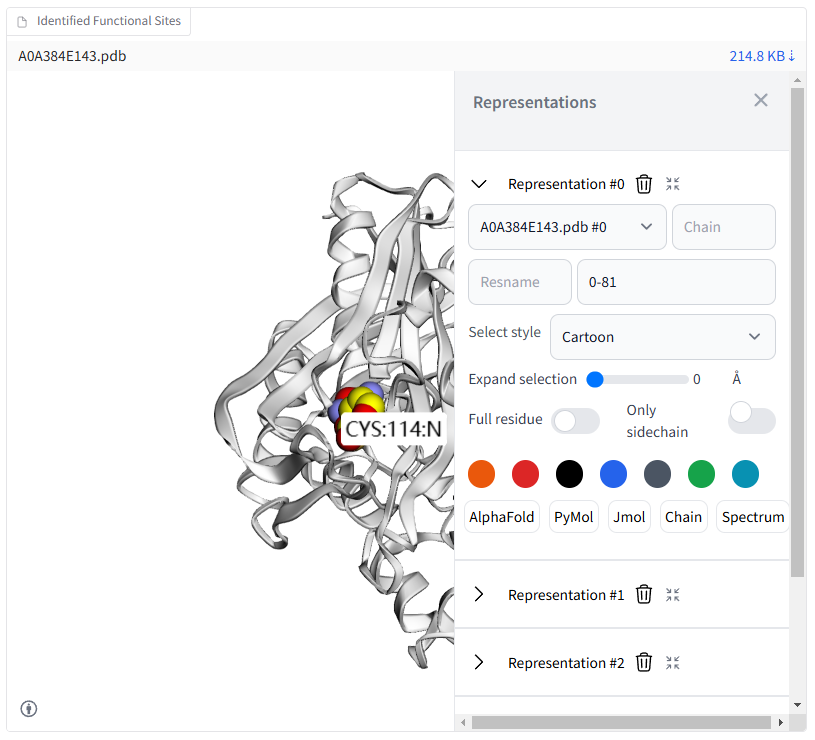


**Supplementary Figure S7. Interactive visualization and analysis of identified active sites.** UniProt ID: A0A384E143.
